# Interactions between light intensity and phosphorus nutrition affect the phosphate-mining capacity of white lupin (*Lupinus albus* L.)

**DOI:** 10.1093/jxb/eru135

**Published:** 2014-04-10

**Authors:** Lingyun Cheng, Xiaoyan Tang, Carroll P. Vance, Philip J. White, Fusuo Zhang, Jianbo Shen

**Affiliations:** ^1^Department of Plant Nutrition, China Agricultural University, Beijing 100193, P. R. China.; ^2^Department of Agronomy and Plant Genetics, University of Minnesota, St. Paul, Minnesota 55108, USA.; ^3^Ecological Sciences Group, The James Hutton Institute, Invergowrie, Dundee DD2 5DA, United Kingdom.

**Keywords:** Photosynthesis, cluster roots, citrate exudation, phosphorus deficiency, sucrose, white lupin.

## Abstract

Light intensity affects photosynthetic carbon (C) fixation and the supply of carbon to roots. To evaluate interactions between carbon supply and phosphorus (P) supply, effects of light intensity on sucrose accumulation, root growth, cluster root formation, carboxylate exudation, and P uptake capacity were studied in white lupin (*Lupinus albus* L.) grown hydroponically with either 200 µmol m^–2^ s^–1^ or 600 µmol m^–2^ s^–1^ light and a sufficient (50 µM P) or deficient (1 µM P) P supply. Plant biomass and root:shoot ratio increased with increasing light intensity, particularly when plants were supplied with sufficient P. Both low P supply and increasing light intensity increased the production of cluster roots and citrate exudation. Transcripts of a phosphoenol pyruvate carboxylase gene (*LaPEPC3*) in cluster roots (which is related to the exudation of citrate), transcripts of a phosphate transporter gene (*LaPT1*), and P uptake all increased with increasing light intensity, under both P-sufficient and P-deficient conditions. Across all four experimental treatments, increased cluster root formation and carboxylate exudation were associated with lower P concentration in the shoot and greater sucrose concentration in the roots. It is suggested that C in excess of shoot growth capabilities is translocated to the roots as sucrose, which serves as both a nutritional signal and a C-substrate for carboxylate exudation and cluster root formation.

## Summary

Increasing light intensity enhances cluster-root formation, citrate exudation, and phosphate-uptake capacity in white lupin under phosphate-deficient conditions by affecting the coordination between P and sucrose signals.

## Introduction

Cluster roots are dense clusters of rootlets with determinate growth that form on lateral roots of many species of Proteaceae and several species in other plant families (Dinkelaker *et al.*, 1995; Skene, 1998; Lamont, 2003; Lambers *et al.*, 2011). White lupin (*Lupinus albus* L.) is an annual legume that forms cluster roots when phosphorus (P) is deficient, and it is frequently used to study the physiology of cluster roots (Keerthisinghe *et al.*, 1998; Neumann *et al.*, 1999; Watt and Evans, 1999; Vance *et al.*, 2003; Lambers *et al.*, 2006). The special morphology of cluster roots increases root surface area for P uptake and this is combined with the release of large amounts of P-mobilizing root exudates (mainly citrate and malate, but also others, such as flavonoids) into the rhizosphere of P-deficient plants (Dinkelaker *et al.*, 1989; Keerthisinghe *et al.*, 1998; Neumann and Martinoia, 2002; Lambers *et al.*, 2006; Weisskopf *et al.*, 2006). These adaptations are considered to be strategies for improving the P-mining capacity of the root system (Lambers *et al.*, 2011; Shen *et al.*, 2013). Root clusters can comprise up to 60% of the whole root system, and the amount of carbon exuded as citrate and malate can range from 10% to greater than 25% of the net fixed carbon (Dinkelaker *et al.*, 1989). Thus, the formation of cluster roots and organic acid exudation are costly carbon investments.

Many studies have been carried out to investigate the contribution of cluster roots to P acquisition in environments with low P availability (Gardner *et al.*, 1983; Watt and Evans, 1999; Neumann and Martinoia, 2002; Shen *et al.*, 2003; Shane and Lambers, 2005). Less attention has been paid to understanding how the environment above the ground influences cluster root formation and function. Light intensity affects photosynthesis, the translocation of carbohydrates to the root, and the growth and morphology of the root system (Hermans *et al.*, 2006; Hammond and White, 2008, 2011). The accumulation of root biomass and total root length is reduced when plants are grown at low light intensities (Aresta and Fukai, 1984; Buttery and Stone, 1988; Demotes-Mainard and Pellerin, 1992; Nagel *et al.*, 2006). Furthermore, studies often indicate that root growth is reduced to a greater extent than shoot growth, and that root:shoot biomass ratios decrease, when plants are grown at lower light intensities (Hébert *et al.*, 2001). Root morphology is also altered by photosynthetic carbon fixation, whether this is affected by changing light intensity (Jensen *et al.*, 1998; Hébert *et al.*, 2001) or carbon-dioxide (CO_2_) concentration in the air (Del Castillo *et al.*, 1989). Photosynthetic carbon fixation has been found to affect cluster root formation (Campbell and Sage, 2002). Higher CO_2_ concentrations in the air increase the number of cluster roots formed by P-deficient white lupin (Watt and Evans, 1999). Exogenous supply of sucrose also stimulates the formation of cluster roots, even in plants grown with a sufficient P supply (Zhou *et al.*, 2008).

Light intensity not only affects root morphology, but also influences the expression of genes that respond to P-deficiency. For example, the expression of *AtPht1;4*, which encodes a P transporter induced by P-starvation, is reduced significantly in roots of *Arabidopsis thaliana* when plants are kept in the dark, but the presence of exogenous sucrose in the growth medium can sustain high expression of *AtPht1;4*, suggesting that the translocation of photosynthates to the roots are important for regulating the expression of this gene (Karthikeyan *et al.*, 2007). This interpretation is consistent with the observations that roots of the *Arabidopsis pho3* mutant, which carries a defective copy of the *SUCROSE TRANSPORTER2* (*SUC2*) gene leading to reduced transport of sucrose from shoot to root, secrete less acid phosphatase than those of the wild-type plant (Zakhleniuk *et al.*, 2001) and that the *Arabidopsis hps1* mutant, in which *SUC2* is overexpressed and the transport of sucrose from shoot to root is greater than the wild-type plant, has a constitutive root P starvation response, even when plants are grown under P-sufficient conditions (Lei *et al.*, 2011). In white lupin, Liu *et al.* (2005) found that the expression of three P responsive genes, *LaPT1*, *LaSAP1*, and *LaMATE*, in cluster roots of plants grown with a low P supply were greatly reduced when plants were transferred to darkness, again suggesting that the supply of photosynthates regulated the expression of these genes.

The main purpose of the experiments described here was to investigate the interactions between light intensity and P availability on the formation of cluster roots. In addition, as the expression of genes encoding phosphoenol pyruvate carboxylase (PEPC) and P transporters in cluster roots is also influenced by both P availability and the supply of carbohydrates to the root (Lejay *et al.*, 2003; Franco-Zorrilla *et al.*, 2005; Liu *et al.*, 2005; Hammond and White, 2008, 2011; Zhou *et al.*, 2008), the expressions of *LaPEPC3*, which is associated with the exudation of citrate, and *LaPT1*, which is associated with P uptake by roots, were also evaluated. Whether greater photosynthesis, and consequently increased translocation of sucrose to the root, was associated with an increased ability of white lupin to acquire P was examined specifically. It is envisaged that an increased ability of white lupin to acquire P might occur by (i) increased C translocation to the root, which results in (ii) increased root sucrose concentration, which (iii) stimulates the production of cluster roots and (iv) the exudation of carboxylates, especially citrate.

## Materials and methods

### Plant material and treatments

Seeds of white lupin (*Lupinus albus* L. cv Kiev Mutant) were germinated on moist filter paper. Four d after germination, seedlings were transferred to pots containing six litres of aerated nutrient solution at a density of five plants per pot. The solution was composed of (µM): Ca(NO_3_)_2_ (2000), K_2_SO_4_ (700), MgSO_4_ (500), KCl (100), H_3_BO_3_ (10), ZnSO_4_ (0.5), MnSO_4_ (0.5), CuSO_4_ (0.2), (NH_4_)_6_Mo_7_O_24_ (0.01), and Fe-EDTA (20). Phosphorus was supplied at 1 µM (P1, deficient conditions) or 50 µM (P50, sufficient conditions) as KH_2_PO_4_. The pH of the solution was adjusted daily to 5.6 using HCl or NaOH. Solutions were changed every three d. Plants were grown in artificially lit controlled environment chambers with 28/18 °C day/night air temperatures, and a relative humidity between 50 and 80%. Two light intensity treatments were employed: low light intensity (200±20 µmol m^–2^ s^–1^) and high light intensity (600±20 µmol m^–2^ s^–1^) during the day. A light intensity of 200 µmol m^–2^ s^–1^ is typical for the cultivation of plants in the laboratory, but it is much lower than natural light intensities found under field conditions. Thus, a light intensity of 600 µmol m^–2^ s^–1^, which approaches natural light intensities, was chosen as the high light intensity treatment.

### Plant harvest and measurements of root morphology

Plants were harvested 28 d after transferring to hydroponics. Plants were either frozen in liquid nitrogen and stored at –80 °C until analysis of gene expression or sucrose concentration, or oven dried at 70 °C for 3 d for assay of dry weight and P concentration. Cluster roots were defined as those portions of primary lateral roots bearing bottle brush-like clusters of rootlets with a density of ten or more rootlets per cm. Root length and root surface area were measured using WinRHIZO (EPSON 1680, WinRHIZO Pro2004b, Canada).

### Determination of net photosynthetic rate, P concentration, and sucrose concentration

Net photosynthetic rate (Pn) was measured on the youngest fully expanded leaf using a portable photosynthesis system (Li6400; LI-COR, Lincoln, NE, USA). Measurements were done between 10.00h and 12.00h.

The concentration of P was determined in root and shoot material after digesting in a mixture of concentrated nitric and perchloric acids. Phosphorus was assayed using the vanado-molybdate method (Westerman, 1990).

Individual samples of leaves and roots were harvested, homogenized, and extracted with 80% ethanol. Sucrose was measured enzymatically in the neutralized supernatant as described by Stitt *et al.* (1989).

### Collection of root exudates and analysis of carboxylates

Root exudates were collected 28 d after transfer to hydroponics. Two hours after the beginning of the photoperiod (8.00–10.00h), intact plants were removed from the hydroponics system and roots were carefully rinsed four times with deionized water to remove ions from the root surface, and then incubated in a trap solution for two hours to collect root exudates. The composition of the trap solution was (µM): MgCl_2_ (200), KCl (100), CaCl_2_ (600), and H_3_BO_3_ (5). The initial pH of the trap solution was 5.6. Following the collection of exudates, solutions were acidified by adding two drops of concentrated H_3_PO_4_ and micropur (Sicheres Trinkwasser, Germany) solution at 0.01g l^−1^ to inhibit the activity of microorganisms. A 10-ml sub-sample of the solution containing root exudates was stored at –20 °C until analysis.

Before analysis, the sub-sample of solution containing root exudates was filtered through sterile Millex GS Millipore 0.22-µm filters. The carboxylates in this solution were analysed by reverse phase HPLC. Separation was conducted on a 250×4.6mm reversed phase column (Alltima C18, 5 Micron; Alltech Associates, Inc., Deerfield, IL, USA) as described by Wang *et al.* (2007). The mobile phase was 25mM KH_2_PO_4_ (pH 2.5) with a flow rate of 1ml min^–1^ at 28 °C and the detection of organic anions was carried out at 214nm.

### Quantitative real-time PCR analysis

Total RNA was isolated and treated with DNase I, and cDNA was synthesized to perform quantitative real time RT-PCR using the SYBR Green PCR Master Mix (Applied Biosystems; P/N4367659) in the iQ5 real-time PCR detection system (Bio-Rad, Hercules, CA) with appropriate primers. Relative quantitative results were calculated by normalization to the lupin tubulin gene.

The primers used to quantify gene expression were: *LaPEPC3*, 5′-CGAACTGTCTATGTGGCGTTGC-3′ and 5′-GAGCCTGTCC CTTACCTCACCC-3′; *LaPT1*, 5′-ATAGTCCAAATTCTGTGTTG GC-3′ and 5′-ATGGTTTTCCCTGCGCCTCTTC-3′; *LaTubulin*, 5′-ACTATCAGCCACCTACTGTTGTTC-3′ and 5′-ACCTTCTTC CATACCCTCACC-3′.

## Results

### Plant growth and biomass allocation

**Fig. 1.**
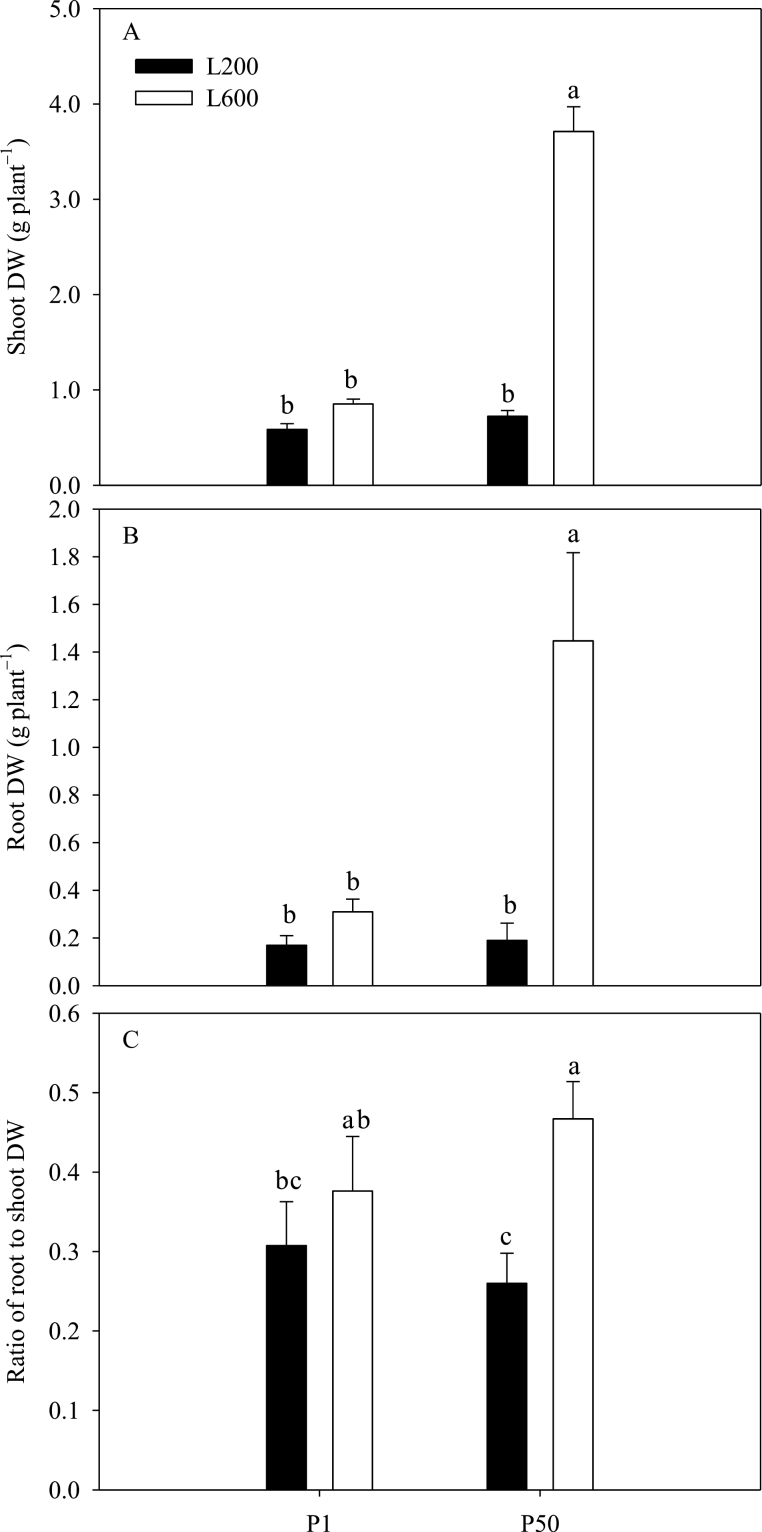
Effects of P supply and light intensity on plant dry weight (DW) (A and B), and on the root:shoot of dry weight ratio (C). Plants were grown in nutrient solution at two levels of P supply (P1=1 µM P, P50=50 µM P) and two levels of light intensity (L200=200 µmol m^–2^ s^–1^, L600=600 µmol m^–2^ s^–1^), and harvested after 28 d growth. Data are averages of three replicates and bars represent standard errors. Data with different letters are significantly different (*P*<0.05).

Shoot and root dry weights more than tripled when the light intensity was increased from 200 to 600 µmol m^–2^ s^–1^ in plants supplied with sufficient (50 µM) P, but were not significantly affected by light intensity when plants were grown at a low P supply (Fig. 1A, B). The root:shoot dry weight ratio was greater in plants grown under high light intensity, but this effect was only significant in the P-sufficient plants, in which increasing light intensity from 200 to 600 µmol m^–2^ s^–1^ almost doubled root:shoot dry weight ratio (Fig. 1C).

### Root development

**Fig. 2.**
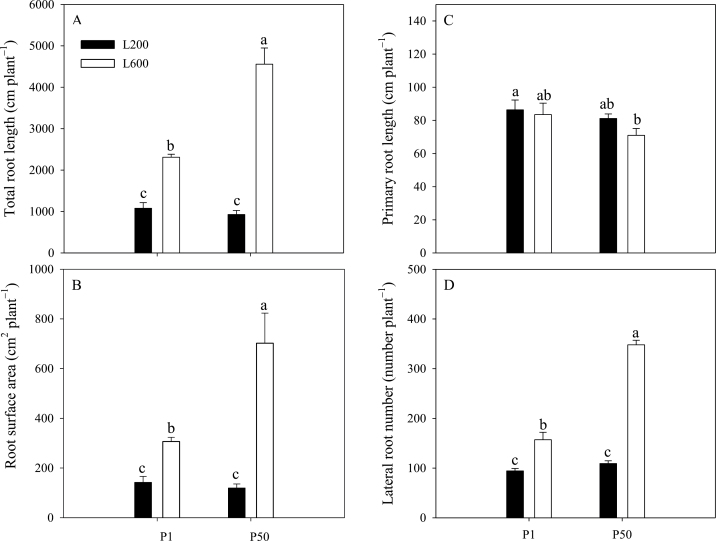
Effects of P supply and light intensity on (A) total root length, (B) root surface area, (C) primary root length, and (D) lateral root number. Plants were grown in nutrient solution at two levels of P supply (P1=1 µM P, P50=50 µM P) and two levels of light intensity (L200=200 µmol m^–2^ s^–1^, L600=600 µmol m^–2^ s^–1^), and harvested after 28 d growth. Data are averages of three replicates and bars represent standard errors. Data with different letters are significantly different (*P*<0.05).

Total root length (Fig. 2A), and, consequently, root surface area (Fig. 2B) were increased significantly by increasing light intensity irrespective of P supply, although the effect was more obvious when plants were grown with an adequate P supply than when they were grown at a low P supply. The increase of total root length was caused by increased lateral root length, as primary root length was unaffected by light intensity at a given P supply (Fig. 2C). The number of lateral roots produced increased with increasing light intensity, especially when plants were P replete (Fig. 2D).

**Fig. 3.**
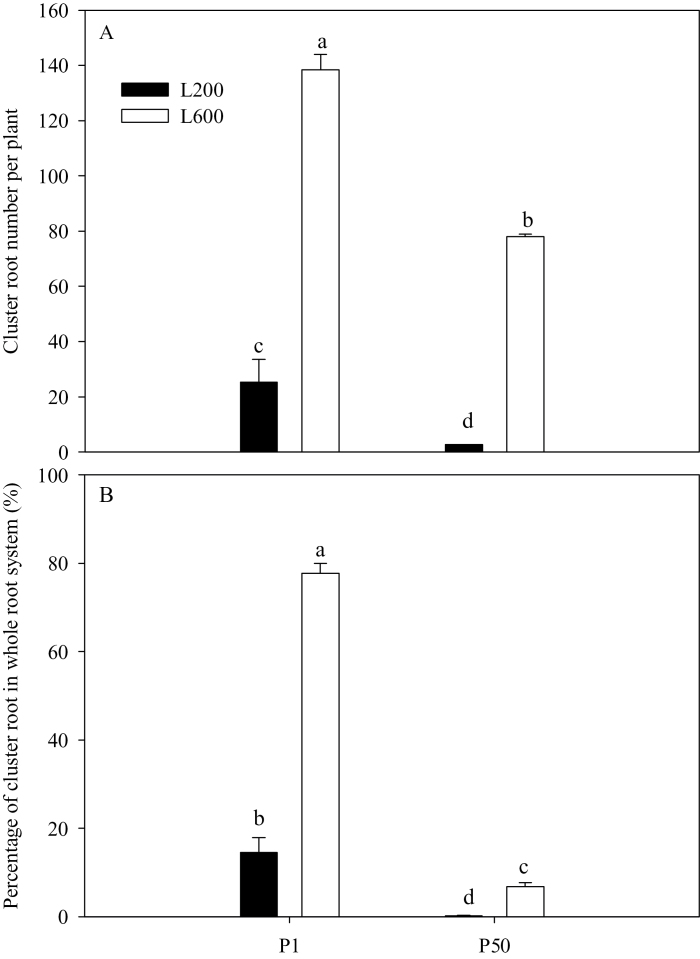
Effects of P supply and light intensity on root formation. Shown are: cluster root number (A) and percentage of cluster root dry weight in whole root system (B) under different treatments. Plants were grown in nutrient solution at two levels of P supply (P1=1 µM P, P50=50 µM P) and two levels of light intensity (L200=200 µmol m^–2^ s^–1^, L600=600 µmol m^–2^ s^–1^), and harvested after 28 d growth. Data are averages of three replicates and bars represent standard errors. Data with different letters are significantly different (*P*<0.05).

The formation of cluster roots was stimulated in plants with an inadequate P supply (Fig. 3), which is consistent with previous studies (Keerthisinghe *et al.*, 1998; Neumann *et al.*, 1999; Watt and Evans, 1999). Increased light intensity also stimulated cluster root formation irrespective of P supply, whether measured as the number of root clusters on a plant or the percentage of total root dry weight biomass allocated to cluster roots (Fig. 3).

### The exudation of carboxylates from roots

**Fig. 4.**
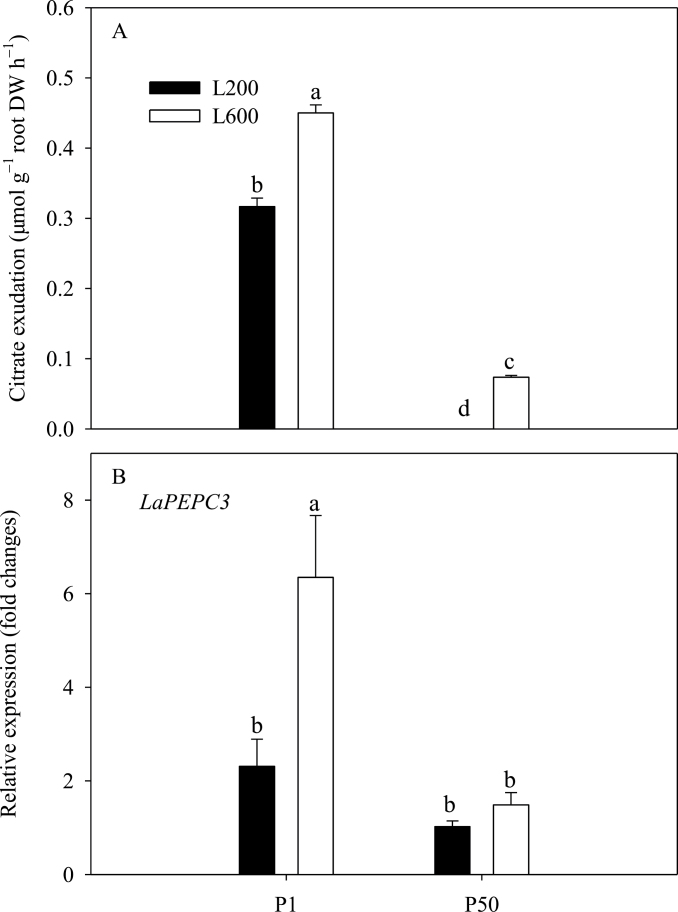
Effects of P supply and light intensity on (A) carboxylate exudation and (B) the expression of *LaPEPC3*. Plants were grown in nutrient solution at two levels of P supply (P1=1 µM P, P50=50 µM P) and two levels of light intensity (L200=200 µmol m^–2^ s^–1^, L600=600 µmol m^–2^ s^–1^), and harvested after 28 d growth. Data are averages of three replicates and bars represent standard errors. Data with different letters are significantly different (*P*<0.05). For *LaPEPC3* expression, data are expressed as relative values based on the expression of *LaPEPC3* in roots of plants grown with P50 and L200 referenced as 1.0. DW represents dry weight.

Roots of plants with a low P supply exuded more citrate than those with an adequate P supply (Fig. 4A). Increasing light intensity from 200 to 600 µmol m^–2^ s^–1^ also increased citrate exudation, even under P-sufficient conditions (Fig. 4A). Increased synthesis of organic acids in the root is accomplished in part by an increase in the activity of phosphoenol pyruvate carboxylase (PEPC), which is induced by P deficiency (Johnson *et al.*, 1996). It was observed that *LaPEPC3*, which might be involved in organic acid synthesis (Peñaloza *et al.*, 2005), was highly expressed under P-deficient conditions (Fig. 4B), and increasing light intensity stimulated the expression of *LaPEPC3* further. These results suggest that the expression of *LaPEPC3* and, consequently, citrate exudation by roots are determined by the interaction of plant P status through P supply, and plant photosynthesis through light intensity.

### Shoot P status and root P uptake capacity

**Fig. 5.**
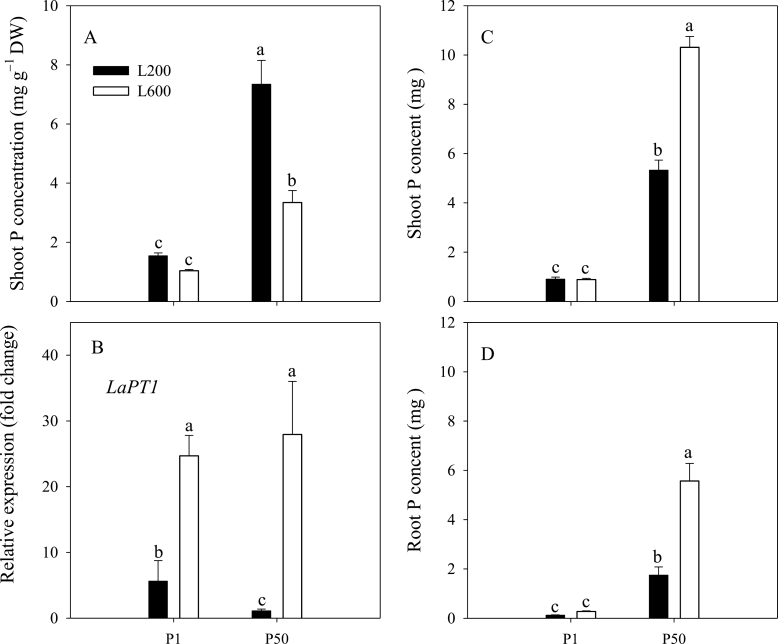
Effects of P supply and light intensity on plant P status (A: shoot P concentration, C: shoot P content, and D: root P content) and the expression of the P transporter gene *LaPT1* (B). Plants were grown in nutrient solution at two levels of P supply (P1=1 µM P, P50=50 µM P) and two levels of light intensity (L200=200 µmol m^–2^ s^–1^, L600=600 µmol m^–2^ s^–1^), and harvested after 28 d growth. Data are averages of three replicates and bars represent standard errors. Data with different letters are significantly different (*P*<0.05). For *LaPT1* expression, data are expressed as relative values based on the expression of *LaPT1* in roots of plats grown with P50 and L200 referenced as 1.0. DW represents dry weight.

Shoots of plants supplied with 50 µM P had greater P concentrations than those of plants supplied with 1 µM P (Fig. 5A). Increasing light intensity decreased shoot P concentration in plants supplied with 50 µM P, but did not cause a decrease in shoot P concentration in plants supplied with 1 µM P. In contrast, the expression of the gene *LaPT1* encoding the phosphate transporter in cluster roots was increased by P deficiency and increasing light intensity (Fig. 5B). However, the expression of *LaPT1* was not simply inversely related to shoot P concentration, and the highest expression of *LaPT1* was observed in plants supplied with an adequate P at high light intensity, which had a higher shoot P concentration than plants grown with a low P supply but showed a relatively low shoot P concentration compared with those plants supplied with 50 µM P under low light intensity. Indeed, high light intensity increased P acquisition significantly when plants were grown with an adequate P supply, as shown by the increased P content of plants grown with higher light intensity under P-sufficient conditions (Fig. 5C, D).

### Photosynthetic efficiency and carbohydrate accumulation in leaves and roots

**Fig. 6.**
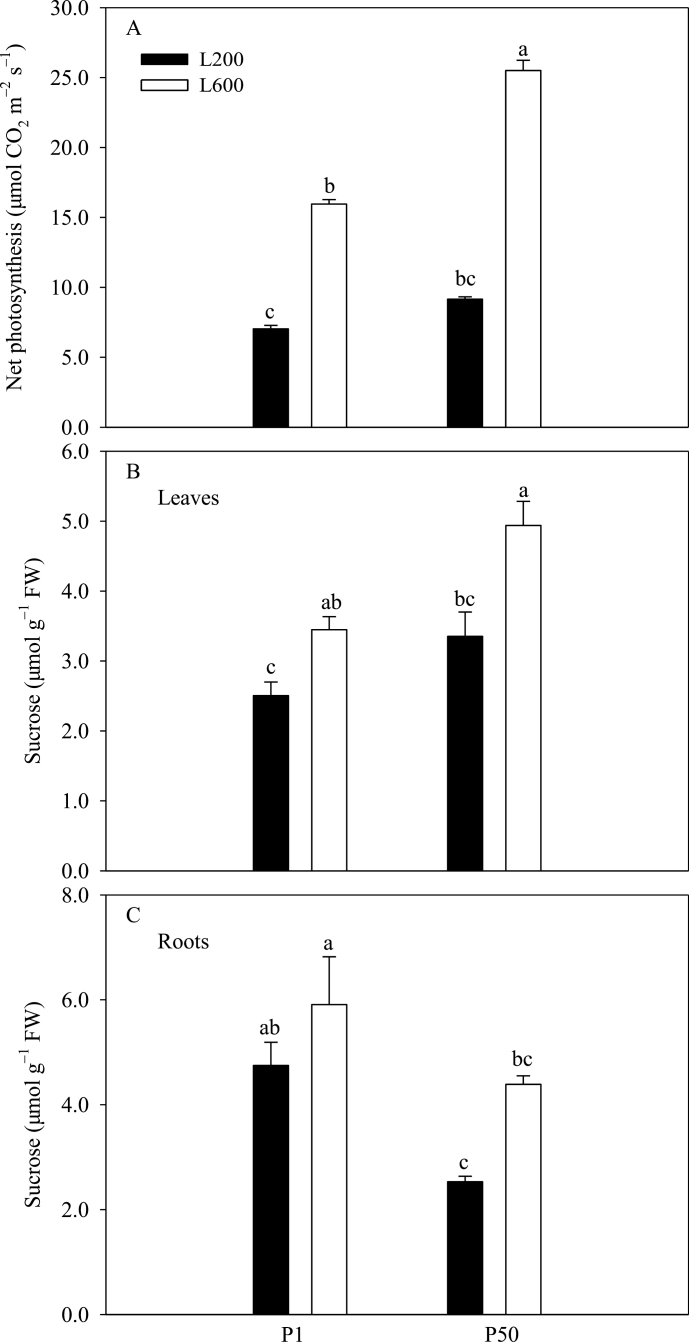
Effects of P supply and light intensity on photosynthetic efficiency (A), and sucrose concentration in leaves (B) and roots (C). Plants were grown in nutrient solution at two levels of P supply (P1=1 µM P, P50=50 µM P) and two levels of light intensity (L200=200 µmol m^–2^ s^–1^, L600=600 µmol m^–2^ s^–1^), and harvested after 28 d growth. Data are averages of three replicates and bars represent standard errors. Data with different letters are significantly different (*P*<0.05). FW represents fresh weight.

Net photosynthesis (Pn) of plants supplied with 1 µM P was lower than those of plants supplied with 50 µM P (Fig. 6A). Increasing light intensity increased the Pn (Fig. 6A) and this effect was greater in plants supplied with more P.

Leaf sucrose concentrations did not differ between plants supplied with 50 µM P or 1 µM P (Fig. 6B). However, sucrose concentrations were much greater in roots of plants supplied with 1 µM P than in roots of plants supplied with sufficient P under lower light condition (Fig. 6C). In addition, sucrose concentrations in both leaves and roots increased with increasing light intensity irrespective of P supply.

### Relationships between cluster-root formation, root exudation, and phosphorus concentration in shoots, or sucrose concentration in roots

**Fig. 7.**
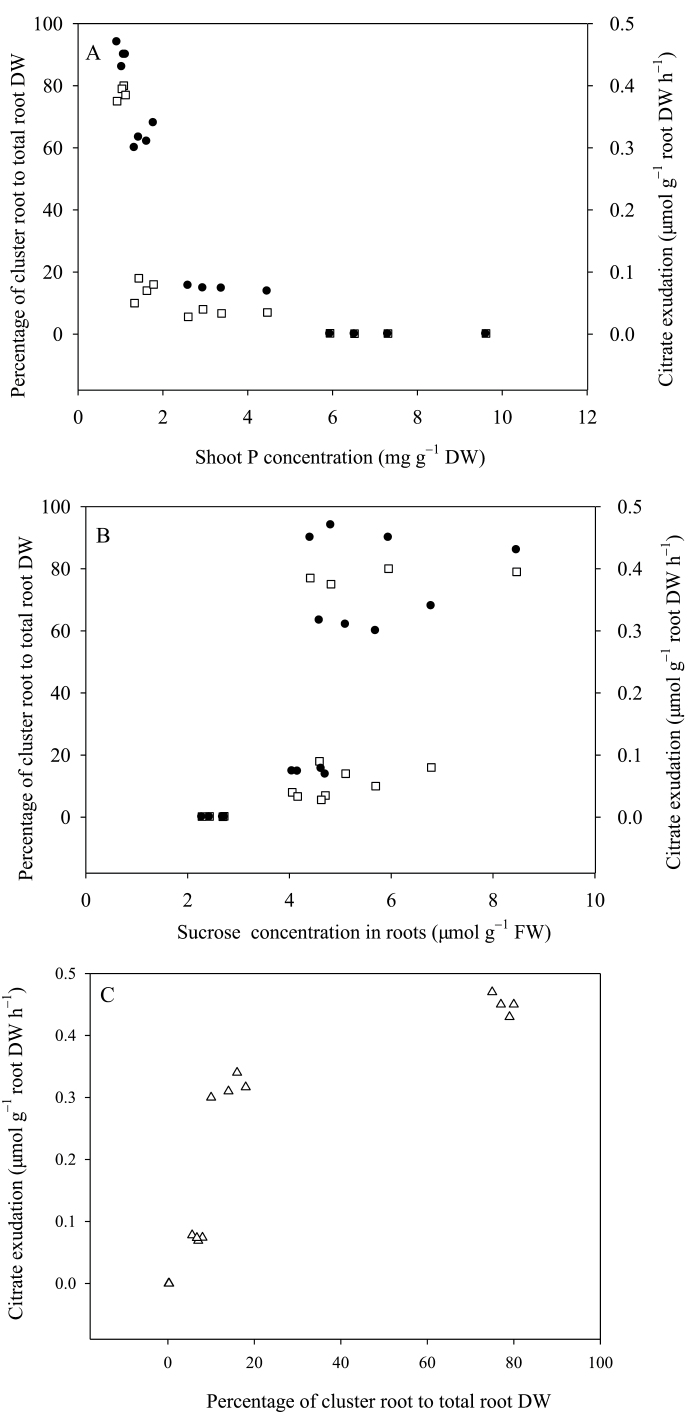
Relationships between cluster root formation (open squares; expressed as percentage contribution of cluster root to total root dry weight (DW)) or citrate exudation (black circles), and shoot P concentration (A) or sucrose concentration in roots (B). (C) Relationship between cluster root formation and citrate exudation. FW represents fresh weight.

The abundance of cluster roots and the exudation of citrate were both inversely related to shoot P concentration (Fig. 7A) and positively related to the sucrose concentration in roots (Fig. 7B). The relationship between cluster root formation and citrate exudation approximated to a rectangular hyperbola (Fig. 7C).

## Discussion

This study was conducted to determine whether greater photosynthesis manipulated by light intensity, and plant P status manipulated by P supply to roots affect cluster root formation and function.

Plants lacking sufficient P show reduced shoot growth rates, translocate C in excess of their shoot growth capability to their roots, and increase their root:shoot biomass ratio to promote P acquisition (Hermans *et al.*, 2006; Hammond and White, 2008). Many previous studies have reported that the increased root:shoot biomass ratio in P-deficient plants is associated with higher carbohydrate concentration in roots (Rychter and Randall, 1994; Ciereszko and Barbachowska, 2000; Hammond and White, 2008, 2011). Similarly, increasing light intensity increases the translocation of C to roots and root:shoot biomass ratio (Wilson, 1988; Cruz, 1997; Hébert *et al.*, 2001). The data reported here are consistent with the observations that both P-deficiency and higher light intensity decrease shoot P concentration (Fig. 5A), and increase root sucrose concentration (Fig. 6C) and root:shoot dry weight ratio (Fig. 1).

In white lupin, P deficiency increases the production of cluster roots and the exudation of organic acids (Keerthisinghe *et al.*, 1998; Neumann *et al.*, 1999; Watt and Evans, 1999; Vance *et al.*, 2003; Lambers *et al.*, 2006). Increasing the supply of carbohydrates to the root also stimulates the production of cluster roots (Watt and Evans, 1999; Zhou *et al.*, 2008). The results of the experiments reported here show that these responses to P deficiency are enhanced by increasing light intensity (Figs 3 and 4). Lupin grown at a light intensity of 600 µmol m^–2^ s^–1^ produced more than twice as many cluster roots as those grown at a light intensity of 200 µmol m^–2^ s^–1^ (Fig. 3), and exuded significantly more citrate (Fig. 4). Increasing light intensity increased both the net photosynthesis and sucrose concentrations in plant tissues (Fig. 6). Based on these observations at least two, non-exclusive, hypotheses might be postulated for the induction of cluster roots by increasing light intensity. Hypothesis A: High light intensity increases photosynthesis, C fixation, and shoot growth rate. Shoot growth rate then exceeds the ability of the roots to supply P to the shoot causing growth-induced P-starvation in the shoot, which produces a systemic signal to induce the formation of cluster roots. Hypothesis B: High light intensity increases photosynthesis, which results in the greater translocation of C to the roots, and the induction of cluster root formation. High light intensity significantly decreased the shoot P concentration of plants supplied with 50 µM P to about 3mg P g^–1^ dry weight (Fig. 5A), which approximates to the critical shoot P concentration for cluster root formation as described by Li *et al.* (2008). Coincidently, P-sufficient plants grown under higher light intensity exhibited P-deficiency responses, including the formation of cluster roots and citrate exudation (Figs 3, 4). Thus, high light intensity could induce P-deficiency in plants supplied with 50 µM P. In addition, a negative relationship was observed between cluster root formation, expressed as the percentage of the total root biomass contributed by cluster roots, and shoot P concentration (Fig. 7A). These results provided evidence in favour of Hypothesis A. Higher light intensity increased the net photosynthetic rate of white lupin grown under both P-deficient and P-sufficient conditions (Fig. 6A). However, photosynthesis was impaired in plants lacking P grown under both low and high light intensities, which is consistent with previous observations on other species (Stitt and Quick, 1989; Rao *et al.*, 1990). The sucrose concentration in roots of plants supplied with 1 µM P was greater than that in roots of plants supplied with 50 µM P irrespective of light intensity (Fig. 6C). Furthermore, the sucrose concentration in roots of plants at a high light intensity was relatively increased in comparison to that at a low light intensity (Fig. 6C), resulting in greater cluster root biomass (Fig. 3B), and exudation of citrate (Fig. 4A). The positive relationship between cluster root formation and sucrose concentration in roots (Fig. 7B) provided additional evidence in favour of Hypothesis B. Both hypotheses are consistent with the data obtained in the experiment reported here.

Shoot-derived carbohydrate signals, and in particular sucrose, have been suggested to play a role in the systemic control of plant P deficiency responses (Hermans *et al.*, 2006; Müller *et al.*, 2007). Adding sucrose to growth media has been found to stimulate the formation of cluster roots of white lupin even when P availability is high (Zhou *et al.*, 2008), and Liu *et al.* (2005) found that the expression of three genes that respond to P supply, *LaPT1*, *LaSAP1*, and *LaMATE*, was reduced in cluster roots of P-deficient white lupin when plants were transferred to the dark. In the experiments reported here, root sucrose concentration was much higher in P-deficient plants than in plants supplied with sufficient P when grown at low light intensity (Fig. 6C). This is consistent with results obtained in bean and soya plants (Fredeen *et al.*, 1989; Ciereszko and Barbachowska, 2000). Reducing light intensity did not affect root sucrose concentration in plants supplied with 1 µM P, which always form cluster roots (Fig 3) and exude citrate (Fig. 4), but reduced root sucrose concentration in plants supplied with 50 µM P, which only form cluster roots (Fig. 3) and exude citrate (Fig. 4) when grown at high light intensity. These observations are consistent with the hypothesis that root sucrose concentration regulates both the formation of cluster roots and citrate exudation of white lupin.

The stimulation of cluster root formation by increased light intensity might not be controlled solely by increased translocation of sucrose to roots, and other systemic signals could also contribute to the induction of cluster root development. For example, auxin has been found to stimulate cluster root formation (Gilbert *et al.*, 2000; Neumann *et al.*, 2000; Skene and James, 2000; Hocking and Jeffery, 2004), and sucrose has been found to enhance the sensitivity of lateral root development to auxin (Jain *et al.*, 2007). During the studies reported here, the expression of an auxin-response repressor gene, which belongs to the Aux/IAA protein family, in roots, was found to be downregulated by high light intensity (data not shown). Earlier experimental evidence also indicates that light can influence auxin movement in shoots (Behringer and Davies, 1992), and Jensen *et al.* (1998) reported that the auxin transport inhibitor NPA inhibits elongation of light-grown but not dark-grown hypocotyls, indicating that light affects auxin transport. The formation of indole-3-acetic acid (IAA) in germinating *Arabidopsis* seedlings was also found to be influenced by light (Bhalerao *et al.*, 2002).

The exudation of citrate increased in white lupin plants lacking sufficient P, as reported previously (Keerthisinghe *et al.*, 1998; Neumann *et al.*, 1999; Watt and Evans, 1999; Vance *et al.*, 2003; Lambers *et al.*, 2006). Increasing light intensity increased citrate exudation, even under P-sufficient conditions (Fig. 4). Although exogenous auxin stimulates the formation of cluster roots in P-sufficient plants, these cluster roots do not show increased citrate exudation, indicating that auxin alone is insufficient to induce the complete response of cluster roots to P-deficiency (Gilbert *et al.*, 2000; Hocking and Jeffery, 2004). Similarly, cluster roots induced by increasing CO_2_ concentration in the air do not exude citrate (Campbell and Sage, 2002). In the study reported here, high light intensity increased both the expression of *LaPEPC3* and the exudation of citrate into the rhizosphere (Fig. 4).

In conclusion, our results demonstrate that increasing light intensity enhances the increase in cluster root formation, citrate exudation, and P uptake capacity induced by P deficiency. It is possible that white lupin integrates the environmental variables of light intensity and P supply, which ultimately determine plant growth, through both the P concentration in shoots and sucrose concentration in plant. It is envisaged that shoot photosynthesis and P status coordinate morphological and physiological responses of roots by translocating C in excess of shoot growth capability to the root. The rate of C translocation to the root, and, specifically, the root sucrose concentration, then serves as both a signal of plant nutritional status, initiating morphological and physiological responses to nutrient imbalance, and as a C-substrate for root growth.
